# Protocol for high-throughput processing of fecal samples for long-read metagenomic sequencing using PacBio HiFi or Oxford Nanopore Technologies

**DOI:** 10.1016/j.xpro.2026.104526

**Published:** 2026-05-08

**Authors:** Jeremiah J. Minich

**Affiliations:** 1 Department of Biology, Baylor University, Waco, TX 76707, USA

**Keywords:** Sequencing, Microbiology, Biotechnology and bioengineering

## Abstract

Long-read sequencing, whether using PacBio (PB) or Oxford Nanopore Technologies (ONT), requires high-molecular-weight (HMW) DNA at high purity and free of contaminants. Here, we present a protocol for high-throughput processing of fecal samples for long-read metagenomic sequencing. We describe steps for microbial inactivation, nucleic acid stabilization, and HMW DNA extraction. We then detail procedures for DNA cleanup, shearing, library preparation, and DNA sequencing.

For complete details on the use and execution of this protocol, please refer to Minich et al.^1^

## Highlights

• Steps for microbial inactivation and nucleic acid stabilization of metagenomic samples
• Instructions for automated extraction of high-molecular-weight DNA from fecal samples
• Guidance on PCR-free library preparation and sequencing optimization

## Abstract (editor highlights)

Publisher’s note: Undertaking any experimental protocol requires adherence to local institutional guidelines for laboratory safety and ethics.

## Graphical abstract

**Figure:**
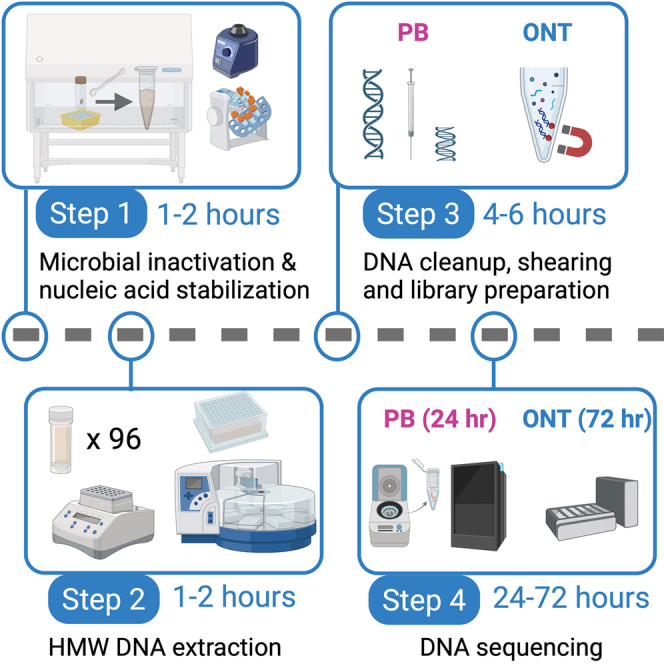

## Before you begin

This protocol describes the specific steps for processing human fecal samples. The fecal samples from the original dataset were all snap frozen in liquid nitrogen and stored at −80 °C. If samples were stored in less favorable conditions and are degraded, its possible they will not perform as well. Although not tested in the original paper, this protocol should work for other sample types including fecal samples from other animals, soil samples, and sediment samples. The key metric being having sufficient HMW gDNA as input for downstream applications.

### Programming and modifying the DNA extraction protocol

**Timing: 30 min**

**Figure 1:**
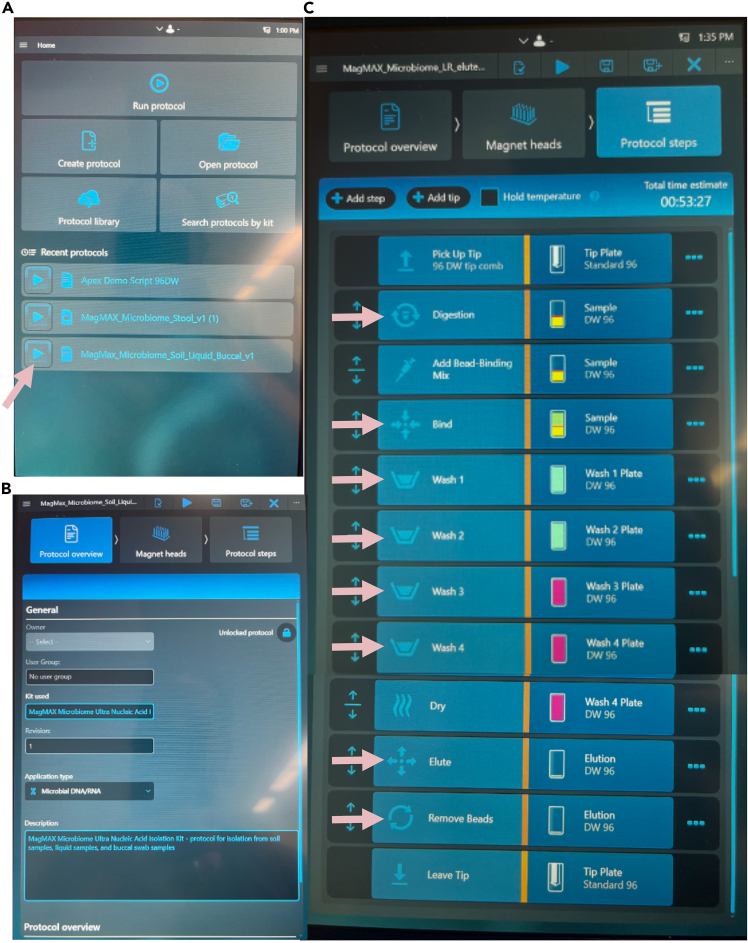
Modifications to the ThermoFisher MagMAX Microbiome protocol to retain HMW microbial gDNA Screen shots of updated protocol as seen from Apex point of view. Change all speeds from medium or fast to slow. (A) overall view of protocol and (B) description of imported protocol. (C) All steps for a given protocol which need to be modified are indicated with pink arrow.

**Figure 2:**
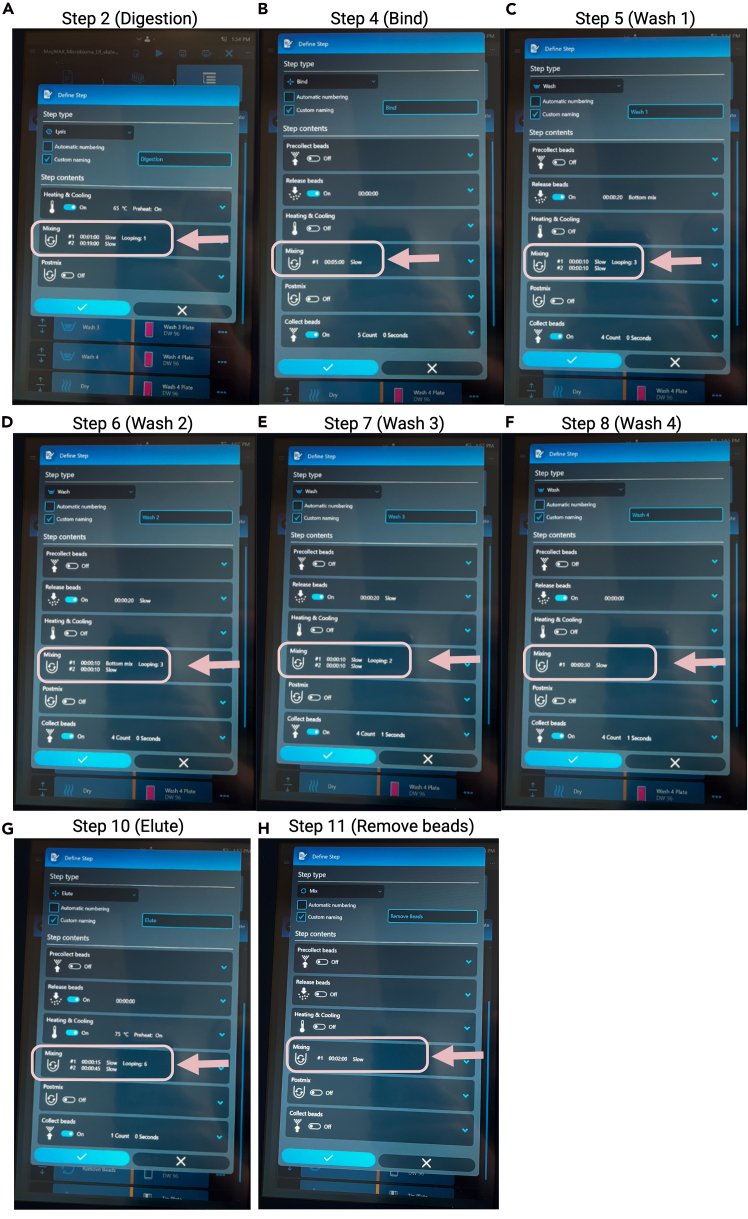
Visualization of steps which require specific modifications to the ThermoFisher MagMAX Microbiome protocol to retain HMW microbial gDNA Figure created with Biorender.com. (A) Step 2: Digestion, (B) Step 4: Bind, (C) Step 5: Wash 1, (D) Step 6: Wash 2, (E) Step 7: Wash 3, (F) Step 8: Wash 4, (G) Step 10: Elute, and (H) Step 11: Remove beads. Specific areas which need to be modified are indicated with a pink arrow and box.
  e. The specific steps which need modified are below (Figure 2):
    i. Digestion step: change the mixing speed from “medium” to “slow”.
    ii. Bind: change from “fast” to “slow”.
    iii. Wash steps 1–4: change from mixing “fast” to “slow”.
    iv. Elute: change from “medium” to “slow”.
    v. Remove bead: change to “slow”.
      ***Note:*** It is very likely that modifications to these steps will result in lower overall DNA extraction yields. If sample yield is critical or challenging to a particular method, it may be useful to try optimizing these steps to improve yields. The elution step would likely be a reasonable starting point: going from slow to medium. Other steps which may help improve yields would include the digestion and binding steps.

1. Download the appropriate scripts for the Kingfisher Flex or Kingfisher Apex.
  a. Go to this website: https://www.thermofisher.com/order/catalog/product/A42358.
  b. Scroll to the bottom of the page – under “Automation Scripts”, download:
     For Apex users:
     KingFisher Apex MagMAX_Microbiome_Soil_Liquid_Buccal_v1.kfx.
     For KingFisher Flex users:
     KingFisher Flex File: MagMAX_Microbiome_Soil_Flex.bdz.
  c. Import the selected method into the KingFisher Apex or Flex instrument.
  d. Make the following modifications to the protocol (see Figures 1 and 2). In short, change all mixing steps where “bottom mix”, “medium”, or “fast” mixes are used to “slow” speed.
    ***Note:*** For the Apex, this can be done on the instrument itself in a GUI interface. For Flex users, you will need to make these modifications using their software and import the updated protocol using a flashdrive.

### Innovation

This protocol introduces three key innovations relative to existing methods. First, it improves sample inactivation and preservation by treating fecal samples with Zymo DNA/RNA Shield followed by extended homogenization on a hula mixer. This buffer inactivates bacteria and viruses, allowing samples to be handled under BSL-1 conditions. Importantly, it also partially lyses cells and deactivates DNases and RNases, preserving the integrity of both DNA and RNA. Effective preservation is especially critical for long-read sequencing, where DNA quality is the primary determinant of success (Minich et al.).^2^ A common weakness in sample preparation occurs during thawing, when reactivated nucleases can rapidly degrade nucleic acids even if initial preservation was adequate. This protocol minimizes degradation during that vulnerable step.

The second innovation is the transition from manual to automated DNA extraction. Automation required substantial optimization of extraction scripts to balance high DNA yield, purity, and minimal shearing. The resulting scripts are provided as part of the protocol, enabling reproducibility while maintaining high-molecular-weight (HMW) DNA suitable for long-read sequencing.

The third innovation occurs during library preparation. This protocol consistently yields long HMW DNA, enabling aggressive 5 kb size selection for PacBio sequencing. In contrast, other protocols often require less stringent size selection, resulting in lower sequencing yields and shorter read lengths. For PCR-free PacBio libraries, a final cleanup using the Qiagen PowerClean kit is critical, likely removing impurities bound to DNA and increasing the number of productive PacBio zero-mode waveguides (ZMWs) during HiFi sequencing. For ONT sequencing, genomic DNA is purified with a 0.4× AMPure cleanup prior to library preparation, improving DNA purity and optimizing fragment size distribution.

### Institutional permissions

**CRITICAL:** Human fecal samples should be obtained using an Institutional Review Board (IRB) approved protocol. Human fecal samples are potentially infectious and should be handled in a Biosafety Level II cabinet using standard aseptic precautions. Animal samples should be obtained using an approved IACUC protocol or similar. Animal fecal samples are generally not considered to be infectious and can typically be handled at the bench in a BSL I laboratory. To be safe however, it is recommended to perform the initial microbial inactivation in a BSL II cabinet. The remainder of the extraction can be done in a BSL I lab environment. All experiments conducted at the time of this protocol development and publication were done in accordance with regulatory standards.

## Key resources table (materials and equipment)

**Table:** 

REAGENT or RESOURCE	SOURCE	IDENTIFIER
**Biological samples**		

Fecal samples	Humans	Depends on study

**Chemicals, peptides, and recombinant proteins**		

Zymo DNA/RNA shield	Zymo Research	Cat#: R1200-125
Ethanol 200 proof 0.125 gallon	Fisher Scientific	Cat#: 04-355-450
Corning Molecular Grade Water	Fisher Scientific	Cat#: MT46000CI

**Critical commercial assays**		

MagMAX Microbiome Ultra kit	ThermoFisher	Cat#: A42358
Qubit dsDNA Broad Range Kit	ThermoFisher	Cat#: Q32850
PacBio HiFi prep kit 96 (includes index plate)	PacBio	PN: 103-381-200
PacBio SMRTbell adaptor index plate A (only needed if buying the SMRTbell prep kit 3.0 ′24 samples’)	PacBio	PN: 102-009-200
AMPure PB beads size selection kit	PacBio	PN: 102-182-500
DNeasy PowerClean Pro Cleanup Kit	Qiagen	Cat#: 12997-50

**Other**		

Filtered P1000 wide bore pipet tips	Fisher Scientific	Cat#: 21-236-2C
Filtered P1000 pipet tips	Fisher Scientific	Cat#: 02-707-461
Filtered P200 pipet tips	Fisher Scientific	Cat#: 02-707-465
spatula (LevGo smartSpatula) micro spatula with sharp spade tip 140 mm	Fisher Scientific	Cat# 18-001-019
2 ml DNA LoBind tubes (Eppendorf)	Fisher Scientific	Cat#: 13-698-792
1.5 ml DNA LoBind tubes (Eppendorf)	Fisher Scientific	Cat#: 13-698-791
DNase and RNase away	Fisher Scientific	Cat#: 21-402-178
Reagent reservoirs 100 ml (BIOTIX)	Fisher Scientific	Cat#: 12-111-100
Reagent reservoirs 5 ml (Fisherbrand)	Fisher Scientific	Cat#: 01-670-462
Free standing microcentrifuge tubes with screw cap (2 ml)	Fisher Scientific	Cat#: 02-682-558
Biological Safety Cabinet	Fisher Scientific	Cat#: 13-201-866
Thermomixer (Eppendorf ThermoMixer C or equivalent) with 1.5 or 2 ml block	Fisher Scientific	Cat#: 05-412-503
KingFisher Flex 96 or Apex extraction robot with 96 DW head	Fisher Scientific	Cat#: 54-009-30
Analytical balance	Fisher Scientific	Cat# 01-804-284
Adjustable tip multichannel pipette (Eppendorf Research plus Move It or equivalent) P1250	Fisher Scientific	Cat#: 05-414-061
Multichannel pipette P1000	Fisher Scientific	Cat#: 05-414-270
Multichannel pipette P200	Fisher Scientific	Cat#: 13-690-049
Tube rack (2) 96 tubes	Fisher Scientific	Cat#: 36-099-3225
Tube rack 80 well (4)	Fisher Scientific	Cat#: 22-313630
Mini Tube Rotator (hula mixer)	Boekel Scientific	Cat#: 260750

## Step-by-step method details

### Bench work preparation

**Timing: 30 min**

1. Sanitize and sterilize the biological safety cabinet by.
  a. Spraying the insides of BSC with 10% bleach and allowing to rest for 10 min.
  b. After 10 min, wipe down using paper towels.
  c. Remove bleach residue using a 70% ethanol spray, and wipe down (BSL-2).
2. Sanitize and decontaminate the benchtop and all equipment (BSL-1) which will be used including tube racks, centrifuge, pipets, etc using the same steps in Step 1.
3. Don a lab coat and/or sleeves to cover one’s skin, and gloves.
  a. At the clean bench, add 280 μL of Zymo DNA/RNA shield 2x buffer to a new screw cap 2 ml tube.
    ***Note:*** This will permit addition of ∼140 mg of sample and will enable one to have enough sample for processing two extractions.
    ***Optional:*** Adjust the volumes of buffer and sample as necessary (if one only has ∼70 mg of sample, then add 140 μL of buffer here and use everything as input into DNA extraction).
  b. Weigh the tube with buffer and record the mass. Using a sharpie, add a number to the lid and side of the tube to indicate tube number.
  c. Print off a sample list with original tube IDs to the respected new tube ID for extraction and tape onto the outside of the biological safety cabinet (BSC) to enable quick reference.
  d. See materials and equipment for preparation of necessary materials.

### Microbial inactivation and nucleic acid stabilization

**Timing: 1**–**2 h per 24 samples; ∼6 h for 96 samples**

**Figure 3:**
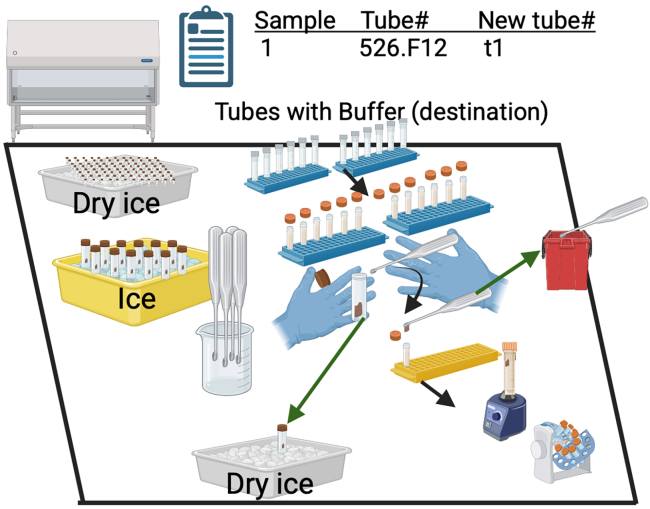
Schematic of fecal sample inactivation and nucleic acid stabilization in Zymo DNA/RNA shield within the Biological Safety Cabinet Figure created with Biorender.com.

See Figure 3 for reference.

4. Place the pre-weighed and pre-aliquoted 2 ml screw cap tubes into the clean BSC.
5. Place the sterile spatulas and a biohazard waste container inside the BSC.
6. Obtain a lab container with ice and one with dry ice.
7. Place all samples to be processed on dry ice to transport from the −80 freezer to the lab area.
8. When ready, place the first 12 samples to be processed on the regular ice to begin thawing. Keep these containers next to the BSC.
9. Move the thawing 12 samples on ice to inside the BSC.
10. Prepare buffered tubes, 12 tubes at a time.
  ***Optional:*** One can do this in higher throughput once comfortable (e.g. 24 tubes at a time) but its important to not let fecal tubes remain on ice for too long as degradation will occur.
  a. Don full PPE (lab coat, safety glasses, sleeves, and gloves) and enter the BSC.
  b. With one’s non-dominant hand, hold the 2 ml screw cap tube containing the DNA/RNA shield.
  c. Unscrew the caps of approximately 12 tubes. Place caps facing down to reduce opportunity for contamination. Ensure tubes are equally spaced (2–3 spaces apart) across 2 tube racks and lids are kept in order.
11. Transfer fecal material to buffered tubes.
  a. From the ice container, remove the first sample, and double check the tube ID with the sample list to confirm it is the correct sample.
  b. With one’s non-dominant hand, hold the screw cap tube containing the fecal sample and twist off the cap.
  c. Using one’s dominant hand, grab a new spatula and gently dig into the tube and obtain approximately 140 mg of feces which is about the size of a pea. If sample is still frozen, hold the tube and wait a min then retry.
  d. Immediately close the tube of the fecal sample and place onto the dry ice while holding the spatula in the dominant hand.
  e. Grab the tube containing buffer of the appropriate sample with the non-dominant hand and carefully dispense the fecal material on the spatula into the tube. Avoid dipping the spatula into the buffer as some of the buffer can adhere to the spatula thereby reducing the volume.
    ***Optional:*** If removing fecal material from the spatula is challenging with a simple tap or scrape, one can alternatively move the intended target tube to a stationary rack and then use a clean, sterile pipet tip to gently scrape or move fecal material off of the spatula into the tube. The risk is that material may accidently miss the tube and fall into the rack or onto the bench. If that happens, be ready to immediately clean the area with bleach and 70% ethanol as previously stated.
  f. Discard the spatula into the biohazard waste bag inside the BSC. Do not reuse – they are contaminated. Discard the biohazard waste, by following institutional policies.
  g. Immediately cap the buffered tube containing fecal material and place in a new rack. Ensure the corresponding tube numbers are correct.
  h. Briefly, vortex for 5 s to ensure that the fecal material is completely immersed in the buffer. Perform gentle flicks if that is not the case.
  i. Repeat this process for the remaining tubes (12 total per batch).
12. Upon completion of the first batch of samples (e.g., 12 tubes), place all 12 tubes onto a hula mixer and allow them to spin at approximately 12 rotations per min (RPM) for a total of 45 min at room temperature.

***Note:*** If too much fecal material was used or if the samples themselves are very clumpy, there is a chance that tubes may need to have additional vortexing, additional buffer, or extended times on the hula mixer. Additional vortexing does not appear to cause DNA fragmentation although we have not tested this extensively. Excessive vortexing, however, may cause shearing of HMW DNA. A lack of homogenization, however, will lead to higher degradation in samples since nucleases are not inactivated promptly.

13. Repeat procedure for remaining 96 samples. Ensure that fecal samples are always put back onto dry ice as soon as possible.
14. Upon completion of homogenization, remove samples from the BSC and weigh the tubes using an analytical balance.
  a. Record the masses.
  b. Calculate the total mass of fecal material per volume (tube final mg – tube initial mg) = total fecal material into the tube. Total volume (280 μL of zymo buffer + mg/ml of feces). Total density: Total mass/Total volume = mg/μL. Perfect would be 140 mg/420 μL = 0.33 mg per μL.

***Note:*** One will typically use 300 μL of this lysate into the DNA extraction step which would be equivalent to ∼99 mg.

15. Sample lysates can be stored at −20 °C or preferability −80 °C until ready for DNA extraction.

***Note:*** Do this process away from the open tubes containing buffer. There is always a chance that when one tries to ‘dig’ out some feces, fecal samples can inadvertently get flung out of the tube.

### High-molecular-weight genomic DNA extraction

**Timing: 2 h**

This is an adapted protocol from the ThermoFisher MagMAX Microbiome Ultra Nucleic Acid Isolation Kit User Guide (Pub. No. MAN0018070 Rev D). All steps can be performed at a regular BSL I facility or benchtop.

This protocol uses the “Soil” version of the protocol which includes a proteinase K incubation. Ensure that the KingFisher Flex or KingFisher Apex is programmed and that the modifications to the protocol for HMW gDNA retention are complete prior to starting the experiment.

#### Before you begin

16. Proceed with setting up all of the plates required for DNA extraction (Step 3 of the protocol as listed above):
  a. Perform these steps in a ultra clean environment such as a dedicated PCR workstation hood which only ever has buffers or preparation reagents used. Ensure this area is both bleached for 10 min followed by a 70% ethanol wipe down.
  b. Use a multichannel pipet and clean reagent reservoirs to transfer reagents to plates.
  c. Ensure 80% ethanol is always made the same day as the experiment and use molecular grade water to reduce chances of contamination.
  d. Setup reagent plates (Part 3 of protocol, page 4).
    i. 4 wash buffer plates.
    ii. 1 elution plate (use 100 μL of elution buffer per well instead of 50 μL and use an elution plate instead of a deep well plate).
    iii. 1 tip comb plate.
17. Create the binding bead mix according to the protocol:
  a. in the same clean space as above (e.g., PCR workstation), add 48 ml of binding solution and 1920 μL (1.92 ml) of total nucleic acid binding beads to a 50 ml conical vial. Set aside. This will be dispensed into the sample plate halfway through the program.

#### Protocol steps

18. Organize all 96 samples from the previous pre-processing steps.
19. If sample lysates were previously frozen, thaw and ensure samples are brought up to room temperature.
20. Print off a plate map indicating which samples will go in which wells. This is useful for when/if mistakes happen. Label lysis tubes and lid with a sharpie (1–96).
21. Lyse microbial cells using a combination of chemical and physical lysis.
  a. Perform the following in replacement of Steps 1–6… Starting on Part 2 of the protocol:
  b. On a clean bench, add 800 μL of lysis buffer to 24 tubes at a time (first 3 columns of the plate).
    ***Note:*** This can be done using an individual P1000 pipet or a P1000 adjustable tip spacing multichannel pipette (e.g. Integra Voyager, Eppendorf Move It) paired with a 100 ml regent reservoir.
  c. Transfer ∼300 μL of lysate to each lysis bead beating tube.
  d. Briefly bump vortex a given screw cap tube from the previous microbial inactivation step to ensure contents are well mixed. Ensure samples are thawed.
  e. Using a P1000 pipet and filtered P1000 wide bore tips, pipet mix the inactivated slurry in the DNA/RNA shield and transfer ∼300 μL into the new lysis tube (containing the 800 μL of lysis buffer.
  f. Tightly screw down the caps of both samples. Return the inactivated lysate tube to a rack and then place back into the freezer for future extractions if lysate remains.
  g. Perform this step for 24 samples.
  h. Place the 24 samples into a Thermomixer and vortex at 2000 RPM at room temperature for 2 min.
    ***Note:*** This is another deviation from the protocol where the original protocol states 10 min at 2500 RPM. Again, for optimizing extraction yield, one could increase the bead beating time or intensity, but it will also likely result in more fragmented gDNA. Additionally, a shorter bead beating time may lead to an underrepresentation of hard-to-lyse bacteria such as spore forming gram positives. We did not verify if increasing the temperature negatively impacts DNA fragmentation or lysis efficiency but this is of course another option for optimization.
  i. Keep at room temperature on a rack and proceed with transferring the other samples.
  j. Repeat Steps 4 – 7 until all 96 samples are prepared for extraction. (total time ∼1 h for 96 samples).
22. Perform proteinase digestion: part 4 of the protocol: (slight modifications).
  a. Add 40 μL of proteinase K to each well of a 96 well Deep well plate. This can be done using a 5 ml reagent reservoir and a P100 or P200 multichannel pipet. Set aside.
  b. Centrifuge tubes at 14,000 *g* for 2 min.
  c. Transfer 500 μL of supernatant to the deep well plate. This can be done sample by sample or using an adjustable tip P1000 multi-channel pipet (8 samples at a time). Avoid any chunks of sample. Repeat spin down if needed.
  d. Start the modified run on the KingFisher Flex or Apex.
23. After 20 min of the run, the instrument will pause and have the user add the binding bead mix.
  a. Vigorously vortex the previously made binding bead mix in the 50 ml conical tube for at least 20 s.
  b. Dispense the mixture into a 50 ml or 100 ml reservoir tray and proceed to add ∼520 μL of this mixture to each sample using a multi-channel pipet. Try to pipet mix a few times to ensure beads are well mixed. Aspirate very slowly as the mixture is highly viscous. Use new pipet tips.
  c. Add plate back onto the robot and proceed.
24. Upon completion of the run, remove the elution plate and cover with a foil adhesion cover as soon as possible. Alternatively, perform quantification for all samples using the Qubit BR dsDNA assay first and then proceed to covering the plate.

***Note:*** QC Metrics explanation. The most important metric is measuring the sample’s DNA concentration using a dsDNA specific method such as the Qubit. It is generally advised to use the Broad Range assay which is more suited for long gDNA according to the manufacturer. Additionally, reading the gDNA quality and quantity with a Nanodrop is useful but not required. The optimal approach for assessing gDNA fragment length is to use the Agilent Femto Pulse which can generate a metric for DNA Quality (DQN). We’ve found however that this metric is not all that predictive of sequencing success thus we do not use this.^1^ A Bioanalyzer or TapeStation gDNA kit can also be used to assess the fragment distribution but both methods struggle to accurately distinguish long or high-molecular-weight (HMW) gDNA. Finally, one can also evaluate gDNA fragment quality by running the sample on a standard 1% gel. If a large smear is observed one should expect to have lower than average fragment sizes during sequencing.

### Library preparation and sequencing with PacBio HiFi

**Timing: 8 h**

25. Follow protocol by PacBio “Preparing whole genome and metagenome libraries using SMRTbell prep kit 3.0” (102-166-600 REV07 JUN2025).

 https://www.pacb.com/wp-content/uploads/Procedure-checklist-Preparing-whole-genome-and-metagenome-libraries-using-SMRTbell-prep-kit-3.0.pdf.

26. Skip Part 1 of the protocol. Do not perform any additional size selection (e.g., using the SRE kit).
27. Perform DNA shearing using the Megaruptor 3. On the original paper, we used the speed 31 at the recommended concentration of 30 ng/μL at 130 μL total volume. The PacBio protocol recommends having at least 500 ng of gDNA. At this stage, it is optimal to use as much gDNA as possible (up to 5 μg). As a rule of thumb, try to use at least 2 μg of gDNA as starting material to maximize success.

***Note:*** We’ve recently determined that manual pipetting at 300x at approximately 1-2 cycles per s may also be sufficient to fragment DNA. This, however, was not part of our original manuscript.

28. Proceed with the rest of the protocol.

***Optional:*** At part 7 (Diluted AMPure PB bead cleanup) the manuscript utilized the more aggressive size selection step (e.g., use 3.1x v/v ‘155 μL’ of resuspended, room-temperature 35% AMPure PB bead solution). This eliminates gDNA less than 5 kb in length. If samples are overly degraded, one can use the less aggressive size selection as indicated (1x SMRTbell bead cleanup).

29. Pooling and final cleanup.
  ***Note:*** The final library conversion calculator is part of the standard PacBio SMRTLink available to users. One will enter the information regarding the concentration, volume, estimated fragment size, and targeted pM value for a library. Assuming a mean fragment size of 10 kb, approximately 163 ng of final library will be required (250 pM) as input to the sequencer using the original chemistry (e.g. Revio or Vega). The SPRQ chemistry, which is only available for the Revio, utilizes a lower input mass. Also, its important to optimize the loading conditions at this stage such as updating the estimate fragment size in future runs based on the mean fragment length of past runs of similar libraries. In practice, its difficult to know the actual fragment size without sequencing. Upon pooling samples, one should aim to have a total of ∼500 ng of library so that one can proceed to doing a final cleanup which removes contaminants inhibiting the polymerase.
  a. Generate a pool of libraries so that a total of 500–1000 ng of library is in a single DNA LoBind tube.
  b. Process this pool using the Qiagen Powerclean Pro kit (Cat# 12997-50). Aim for a 30–50% recovery of sample.
  c. Quantify the remaining gDNA using the Qubit dsDNA BR or HS kit.
  d. Proceed to Part 8 of the PB protocol (ABC binding).

### Library preparation and sequencing with Oxford Nanopore Technologies

**Timing: 6 h**

30. Follow steps for the Native Barcoding Kit 24 or 96 SQK-NBD114.24 or NBD114.96).
31. The only modification to this protocol is to perform a 0.4 x AMPure bead cleanup of each gDNA sample prior to starting the library preparation.
  a. Add 0.4x volume AMPure XP beads to the gDNA.
  b. Pipet mix 10x and incubate for 10 min at room temperature.
  c. Place on a magnet and allow to become clean, remove supernatant.
  d. Keep sample on the magnet and wash beads with 80% ethanol a total of two times.
  e. Remove any residual ethanol by removing tube or strip tube from the magnet, briefly spin down and place back onto the magnet. Pipet off remaining ethanol.
  f. Remove samples from the magnet and resuspend in either DNase and RNase free water or elution buffer. Pipet mix 10x and allow to incubate at room temperature for 10 min to elute DNA.
  g. Place sample back onto the magnet, remove and retain supernatant.
  h. Quantify the DNA recovery and proceed to the library preparation for ONT.

## Expected outcomes

Based on our previous results, one can expect to obtain approximately 6.154 μg of gDNA per sample (∼61 ng/μL at 100 μL elution volume) when using ∼100 mg of feces as input.^1^ There will be variations however which is often driven by the variance in sample quality. Watery stools for instance, may generate lower yields. DNA should be of sufficient quality to yield at least 1 library preparation using the SMRTbell3.0 protocol for PacBio and ONT.

## Limitations

To date, this protocol has only been verified on human fecal samples which were preserved initially in liquid nitrogen and stored at −80. This indicates that the original sample quality would have been very high. Many studies may not have fecal samples stored in such quality conditions. Thus, its possible that samples stored in less tedious manners such as held at room temperature for extended amounts of time or kept in 4 C or −20 may have higher levels of sample degradation leading to suboptimal performance. This approach has been tested on rainbow trout fecal samples with good success. These samples however, were processed by placing feces directly into the DNA/RNA shield, held at room temperature for up to 2 h and then stored in the −80. There is no reason, however that the protocol would fail for other fecal sample types or even other microbiome types. It has not been tested for soil or sediment but its reasonable to believe it would work for that.

## Troubleshooting

### Problem 1

The most significant and obvious failure would be low DNA yields whereby there is insufficient DNA to proceed with library preparation.

### Potential solution

To address this, there are a few tweaks to the protocol. First, one should try to increase the lysis time on the vortexing stage from 2 min to 4 min. This should result in increased DNA yield albeit with more fragmented DNA. Another potential approach is to increase the pre-lysis stage whereby the incubation in the nucleic acid stabilization buffer is increased. If samples are very challenging, it is recommended to consider having some positive controls which are processed alongside and even as a spike in to the samples of interest to help determine where the problems are occurring.

### Problem 2

DNA quantity appears sufficient for library preparation but either the library preparation reactions have very low yield or sequencing yield (HiFi) is lower than expected.

### Potential solution

If sequencing yields are low, its possible that the amount of DNA used as the library input was also insufficient. Often times this can be based on an incorrect assumption of the average fragment size. For HiFi sequencing, a fragment or insert size chosen should be between 5000–10000 bp. Once sequenced, one can use the mean read length as a baseline for future calculations. Another potential problem with PCR free libraries is the carry-over of contaminants or inhibitors which bind to DNA and prevent the HiFi polymerase from going around the template. If carry over contamination is suspected or if the DNA quality is insufficient, it is important to go back and try to quality check the DNA using a Nanodrop. The 260/230 and 260/280 values can be important metrics to assess quality assuming there is sufficient DNA. It is recommended to always perform a pilot experiment on new sample types which are suspected to have questionable quality. Again, one can do this at the DNA extraction stage by including a 1) sample, 2) control, and 3) sample spiked with control. One can also do this as the DNA stage using the same approach. Both methods can help ascertain whether the problem occurs at the DNA extraction stage or the library preparation stage.

## Resource availability

### Lead contact

Further information and requests for resources and reagents should be directed to and will be fulfilled by the lead contact, Jeremiah J. Minich (jake_minich@baylor.edu).

### Technical contact

Technical questions on executing this protocol should be directed to and will be answered by the technical contact, Jeremiah J. Minich (jake_minich@baylor.edu).

### Materials availability

This study did not generate any new unique reagents.

### Data and code availability

The scripts for the protocol are included in Figure 1.

## Acknowledgments

The NOMIS Postdoc fellowship supported J.J.M. The development of this protocol was made possible by the use of fecal samples shared by Dr. Kevin Stephenson, Dr. Mark Manary, and Dr. Kenneth Maleta.

## Author contributions

J.J.M. performed all protocol optimizations, developed the final version of the protocol, and wrote the manuscript.

## Declaration of interests

The author declares no competing interests.
